# Pharmacokinetic overview of ethinyl estradiol dose and bioavailability using two transdermal contraceptive systems and a standard combined oral contraceptive 

**DOI:** 10.5414/CP202064

**Published:** 2014-10-08

**Authors:** Birte Hofmann, Isabel Reinecke, Barbara Schuett, Martin Merz, Christian Zurth

**Affiliations:** Bayer Pharma AG, Berlin, Germany

**Keywords:** transdermal female contraceptive patch, pharmacokinetics, gestodene, ethinyl estradiol, bioavailability

## Abstract

Objective: To determine the relative bioavailability of ethinyl estradiol (EE) and gestodene (GSD) after application of a novel transdermal contraceptive patch vs. a standard combined oral contraceptive (COC) pill (study 1), and to evaluate the pharmacokinetics (PK) of EE after application of the EE/GSD patch compared with an EE/norelgestromin (NGMN) patch (study 2). Materials: Participants were healthy, non-obese women aged 18 – 45 years (study 1) or 18 – 35 years (study 2). Compositions of study treatments were as follows: 0.55 mg EE/2.1 mg GSD (EE/GSD patch); 0.02 mg EE/0.075 mg GSD (standard COC); 0.6 mg EE/6 mg NGMN (EE/NGMN patch). Methods: In study 1, which consisted of 3 treatment periods (each followed by 7 patch- or pill-free days), treatments were administered in one of two randomized orders: either P–M–E (EE/GSD patch (P) every 7 days for 28 days → COC (M) once-daily for 21 days → two 7-day patch-wearing periods followed by one 10-day patch-wearing phase (E)), or the same treatments administered in sequence M–P–E. For study 2, participants received either the EE/GSD patch or EE/NGMN patch for seven treatment cycles (one patch per week for 3 weeks followed by a 7-day patch-free interval). Results: In study 1, average daily exposure to EE was similar for treatments P and M; the mean daily area under the concentration-time curve (AUC) ratio of treatment P vs. treatment M for EE was 1.06 (90% confidence interval (CI): 0.964 – 1.16), indicating average daily delivery similar to oral administration of 0.019 – 0.023 mg EE. For unbound GSD, average daily exposure was lower for treatment P vs. treatment M. The mean AUC ratio of treatment P vs. treatment M for unbound GSD was 0.820 (90% CI: 0.760 – 0.885), indicating average daily delivery from the patch of 0.057 – 0.066 mg GSD. Prolonged patch wearing did not result in a distinct decline in GSD and EE serum concentrations. In study 2, AUC at steady state (AUC_0–168,ss_), average steady-state serum concentration, and maximum steady-state serum concentration for EE was 2.0 – 2.7-fold higher for the EE/NGMN patch vs. the EE/GSD patch. The EE/GSD patch was well tolerated in both studies. Conclusions: Based on the 90% CI of the AUC ratio of oral treatment vs. patch application for unbound GSD and EE, the daily doses of GSD and EE released from the EE/GSD patch over the 7-day application period provided the same systemic exposure as those recorded after daily oral administration of a COC containing 0.02 mg EE and 0.06 mg GSD. The EE/GSD patch showed reduced EE exposure compared with the EE/NGMN patch. Together with its good tolerability, these properties support the EE/GSD patch as an effective and well-tolerated alternative to available transdermal and oral contraceptives.

Clinical trials registration no.: NCT00984789, EudraCT no.: 2009-016972-66, EUDRA CTno.: 2008-007308-27; NCT00984789 

## Introduction 

The systemic delivery of steroid hormones through the transdermal route is a well-established treatment for post-menopausal women, using patches that contain estrogen either alone or in combination with a progestin [1]. Transdermal delivery of hormones is also used effectively for contraception, and, in Europe, a transdermal contraceptive patch was approved in 2002. This patch releases ethinyl estradiol (EE) and norelgestromin (NGMN) over a 7-day period, providing the same systemic exposure as recorded after daily oral administration of a combined oral contraceptive (COC) containing 0.0339 mg EE and 0.203 mg NGMN [2].* 

*In the US, a slightly different formulation was approved by the Food and Drug Administration in November 2001. 

Daily use of COCs represents the most common form of contraception used by women in the developed world [3], and daily COCs are highly efficacious when used correctly. However, it is important to recognize that poor compliance is common with daily COCs and can greatly reduce contraceptive efficacy, leading to unwanted pregnancies [4]. In addition, oral administration of contraceptive hormones may result in large fluctuations in serum hormone concentrations [5], with large intra- and inter-individual pharmacokinetic (PK) variations in serum concentrations [6], while EE bioavailability is low (38 – 48%) [5, 7]. 

In recent years, a novel, once-a-week, transparent, transdermal contraceptive patch has been developed, which may have benefits over COCs (Bayer Pharma AG, Berlin, Germany, unpublished data). Transdermal contraceptives offer several advantages over COCs, including effective hormone absorption and the provision of relatively constant serum concentrations [5, 8, 9]. When considered alongside the convenience of weekly patch application rather than daily oral administration, transdermal contraceptives widen contraceptive choice and may increase levels of compliance. 

Both EE and the progestin gestodene (GSD) are well absorbed through the skin and are, therefore, appropriate agents for inclusion in a transdermal contraceptive formulation [5, 8, 10]. EE is the most potent estrogen agonist currently available [11], and its use in COCs is well documented [6], while GSD is an extensively researched progestin with an established efficacy and safety profile that has been used widely as a contraceptive agent in Europe for over 2 decades [8, 12, 13]. In addition, only a low dose of GSD is required, which allows for a small patch size (Bayer Pharma AG, unpublished data). 

In this paper, we report the results of two studies that examined the PK profile of a novel transdermal contraceptive patch containing 0.55 mg EE and 2.1 mg GSD. The first study aimed to determine the relative bioavailability of these hormones after application of the EE/GSD patch in comparison with a standard COC containing 0.02 mg EE and 0.075 mg GSD. In the second study, the PK of EE following application of the EE/GSD patch was compared with that of a transdermal contraceptive patch containing 0.6 mg EE and 6 mg NGMN (marketed in Europe as EVRA^®^). The latter assessment was based on sparse blood sampling in a large Phase IIIa trial (Gruber et al., manuscript in preparation) and the application of a population PK approach to describe the PK characteristics of EE. Additionally, also the PK characteristics of GSD for the EE/GSD patch were studied. 

## Methods 

### Design 

Study 1 was an open-label, randomized, intra-individual, crossover phase I study in healthy women. The primary objective was to compare the relative bioavailability of EE and GSD from the EE/GSD patch with that of a standard oral COC containing 0.02 mg EE and 0.075 mg GSD (Meliane^®^, Bayer Pharma AG, Berlin, Germany) and to estimate the dose delivery of EE and GSD from the EE/GSD patch. A secondary objective was to assess serum concentrations of EE and GSD when the patch was worn for an additional 3 days beyond the designated week. The study comprised 6 periods: screening, pre-dose, treatment period 1, treatment period 2, treatment period 3, and follow-up. Each woman received both contraceptive formulations in one of two orders: either treatment P (the EE/GSD patch applied once every 7 days for 28 days followed by a 7-day patch-free interval), followed by treatment M (COC once-daily for 21 days followed by a 7-day pill-free interval), followed by treatment E (two 7-day patch-wearing periods followed by one 10-day patch-wearing phase and a 7-day patch-free interval), referred to here as sequence P–M–E; or the same treatments administered in sequence M–P–E. The duration of treatment P was 28 days, rather than the 21 days envisioned in practice, to ensure steady-state serum concentrations of EE, GSD, and sex hormone-binding globulin (SHBG) at the time of PK analysis. Patches were applied to the lower abdomen. 

Study 2 was a multi-center, open-label, randomized, comparative phase IIIa study comprising two parallel treatment arms, with women receiving one of two transdermal contraceptives (the EE/GSD patch or the EE/NGMN patch). Treatment consisted of 7 consecutive treatment cycles (one patch per week for 3 weeks followed by a 7-day patch-free interval). The primary objective was to investigate the bleeding pattern and cycle control parameters of the EE/GSD patch compared with a transdermal patch containing 0.6 mg EE and 6 mg NGMN; these data will be reported elsewhere. Secondary objectives, reported here, included a comparison of the PK of EE following application of either the EE/GSD patch or EE/NGMN patch, a description of the PK of EE, SHBG, and GSD following application of the EE/GSD patch for up to 7 treatment cycles using a population PK approach, a covariate analysis of the influence of demographic and physiologic covariates (e.g., body weight and application-site effect) on the PK of EE and GSD, and an examination of compliance and adverse events (AEs). 

Written informed consent was obtained from all participants in both studies. The studies were conducted in accordance with the ethical principles set out in the Declaration of Helsinki and the International Conference on Harmonization of Technical Requirements for Registration of Pharmaceuticals for Human Use Guideline E6: Good Clinical Practice. Both studies were approved by the individual Ethics Committees of the participating sites. 

### Assessments 

In study 1, blood samples for treatment P were taken pre-treatment and before patch application on days 8, 15, and 22. Additional samples were taken on days 22 – 29 at various points after application of the fourth patch, then on days 29 – 34 after removal of the last patch. For treatment M, blood samples for PK analysis were taken pre-treatment and before pill administration on days 8, 15, 18, and 21. Additional samples were taken between days 21 and 26 after last intake. For treatment E, blood samples were taken pre-treatment and before patch application on days 8 and 15. Additional samples were taken between days 15 and 25 after application of the third patch, and between days 25 and 30 after removal of the third patch. The concentrations of EE and GSD in serum were determined by a validated liquid chromatography/tandem mass spectrometry method, with lower limits of quantification of 2.5 and 50 ng/L, respectively. The concentration of SHBG was determined by a commercially available validated immunoassay method (AutoDELFIA^®^, Perkin Elmer Life and Analytical Sciences, Turku, Finland) with a lower limit of quantification of < 10 nmol/L. The accuracy and precision of the analyses of EE, GSD and SHBG were monitored using appropriate quality control samples. These analyses were carried out under the supervision of the sponsor’s Drug Metabolism and Pharmacokinetics department. The calibration range of the procedure for determination of EE was 2.49 – 124.30 ng/L upper limit of quantification (ULOQ); lower limit of quantification (LLOQ) was set to 2.50 ng/L. Mean inter-assay accuracy of back-calculated concentrations in calibrators was 99.36 – 101.00%, and precision was ≤ 4.23%. Quality control samples in the concentration range 7.46 – 93.23 ng/L were determined with an accuracy of 98.69 – 101.00% and a precision of 2.62 – 4.08%. The calibration range of the procedure for determination of GSD was 0.04958 – 19.832 µg/L (ULOQ) and the LLOQ was set to 0.050 µg/L. Mean inter-assay accuracy of back-calculated concentrations in calibrators was 98.13 – 102.57%, and precision was ≤ 5.88%. Quality control samples in the concentration range 0.14874 – 14.874 µg/L were determined with an accuracy of 99.43 – 102.17% and a precision of 2.40 – 5.05%. The unbound concentration of GSD was calculated based on the total concentration of GSD and the respective concentration of SHBG and albumin. The primary PK variable was the average daily area under the concentration–time curve (AUC) for EE and unbound GSD – treatment P: AUC from 0 to 168 hours after application of the fourth patch (AUC_0–168_) divided by 7; treatment M: AUC from the last pill intake until 24 hours thereafter (AUC_0–24_). Secondary PK variables included AUC from 0 to 240 hours after application of the third patch (AUC_0–240_) for treatment E, and the maximum and average serum concentrations (C_max_ and C_av_, respectively) during week 4 for treatment P, day 21 for treatment M, or week 3 for treatment E. AEs were also monitored. 

In study 2, blood samples for PK assessment were taken at any time during pre-treatment, plus two samples obtained at any time during the patch-wearing periods (i.e., treatment cycles 1 – 7), preferably in treatment cycles 3 and 7. Two further samples were taken during the patch-free interval of these treatment cycles, preferably within 12 hours after patch removal (day 22) for treatment cycles 3 and 7 at 1-hour intervals. Concentrations of SHBG, EE, and GSD in serum were assessed using the methods described for study 1. The accuracy and precision of the analyses of SHBG, EE, and GSD were monitored using appropriate quality control samples. 

The population PK analysis focused on characterization of the PK of EE and GSD, including the interaction with SHBG, for women in the EE/GSD patch group, and characterization of the PK of EE for women in the EE/NGMN patch group. PK data were analyzed using non-linear mixed-effects models [14]. These models include fixed effects describing the typical values (population means), and random effects describing the variability of fixed-effects parameters within the study population (inter-individual variability and residual error). First, a population PK model was developed for the PK of EE, using data for the EE/GSD patch and the EE/NGMN patch (“population PK model EE”). Following this, a population PK model was developed for the PK of EE and GSD, including the interaction with SHBG, fixing the parameters of EE (fixed effects) to those of “population PK model EE” and using EE, GSD, and SHBG data for the EE/GSD patch (“population PK model EE/SHBG/GSD”). Based on these models, individual PK parameters, such as AUC_0–168_ at steady state (ss), average drug concentration (C_av,ss_), and maximum drug concentration (C_max,ss_), were estimated. In addition to the PK analyses, AEs were monitored throughout the study. A visual predictive check was performed in order to assess whether the final population PK model adequately described the observations to fit the purpose of predicting the exposure. For each subject, the serum concentration–time profiles were simulated 500 times by means of Monte Carlo simulation. Briefly, in a Monte Carlo simulation, random values are drawn from the distributions of the identified random effects. The 5^th^, 50^th^, and 95^th^ percentiles of the predictions were then calculated and compared with the observations. 

### Statistical analysis 

Study 1 was an exploratory study, thus, no formal determination of sample size was carried out. Summary statistics for PK parameters included the geometric mean, coefficient of variation (CV), range, and number of measurements. The relative bioavailability of the EE/GSD patch was assessed vs. a standard COC by analyzing EE, unbound GSD and total GSD in terms of AUC_0–168_ divided by 7 for treatment P and AUC_0–24_ for treatment M, assuming log-normally distributed data. Logarithms of these characteristics were studied by analysis of variance (ANOVA), including sequence, subject (sequence), period, and treatment effects. Based on these analyses, point estimates (least square (LS) means) and exploratory 90% confidence intervals (CIs) for the patch : pill ratio of PK parameters was calculated by re-transformation of the logarithmic results given by the ANOVA using the intra-individual standard deviation (SD). The 90% CIs were used to estimate the doses of EE and GSD delivered by the patch, by multiplying the interval limits by a factor of 20 for EE and a factor of 75 for GSD, respectively (based on the COC formulation 0.02 mg EE and 0.075 mg GSD). 

In study 2, all variables were analyzed using descriptive statistical methods. As the analyses were purely descriptive, no formal sample size calculation was undertaken. Statistical evaluations were performed by using the software package SAS release 9.1 or higher (SAS Institute Inc., Cary, NC, USA). 

## Materials 

In study 1, treatments P and E both used the EE/GSD patch containing 0.55 mg EE and 2.1 mg GSD. Patches were applied and removed under supervision of a member of the investigator’s team between 6:00 AM and 10:00 AM. Treatment M was a COC pill containing 0.02 mg EE and 0.075 mg GSD, to be taken daily between 6:00 AM and 10:00 AM. All women kept a diary detailing intake of pills and patch application. 

In study 2, participants were randomized 1 : 1 to use one of two transdermal contraceptive patches: either a patch containing 0.6 mg EE/6 mg NGMN (equivalent to a daily oral dose of 0.0339 mg EE/0.203 mg NGMN), or the EE/GSD patch containing 0.55 mg EE/2.1 mg GSD. Patches were applied to the upper arm, abdomen, or buttocks. Treatment compliance and patch adhesion were evaluated based on participant diaries, with women asked to record application deviations (defined as patch loss for ≥ 24 hours or patch replacement delayed for ≥ 48 hours), the reason for patch removal, and the dates they did not wear a patch. 

## Subjects 

Participants were healthy, non-obese (body mass index: 18 – 30 kg/m^2^) women, aged 18 – 45 years (study 1) and 18 – 35 years (study 2). In the latter study, women were not using any other methods of contraception. Exclusion criteria for both studies included: a positive pregnancy test; any disease, condition, or drug use that could affect the PK of the study agents or be worsened under hormonal treatment; misuse of alcohol/drugs/medicines; or delivery, abortion, or lactation in the previous 3 months; undiagnosed abnormal genital bleeding; or hypersensitivity to any ingredient of the study drug or significant skin reaction to transdermal preparations. Additional exclusion criteria for study 1 included: contraindications for use of hormonal contraceptives (e.g., history of venous/arterial thromboembolic disease); clinically relevant findings in the physical or gynecologic examination or standard laboratory tests or vital sign measurements; smoking for women aged 31 – 45 years (or smoking > 10 cigarettes/day for women aged 18 – 30 years). Participants in study 1 were also required to accept a synchronizing cycle for the purpose of study logistics, during which they were required to use a hormonal contraceptive for 15 – 42 days and then cease use 7 days before the first treatment. 

## Results 

In study 1, a total of 25 women was randomized; 13 to the treatment sequence P–M–E and 12 to the sequence M–P–E. Baseline characteristics of these participants are shown in Table 1. 20 participants completed all 3 treatment periods and 5 discontinued the study (1 in period 1, 2 in period 2, and 2 in period 3). Of the 25 randomized and treated participants, 3 had major protocol deviations, all of which were categorized as treatment deviations. All major protocol deviations were due to premature study discontinuation. Therefore, 22 participants were evaluated in the PK analysis (11 per treatment sequence), and 25 participants were evaluated in the safety analysis. 

In study 2, a total of 398 women were randomized and received study treatment, with 245 (EE/GSD, n = 118; EE/NGMN, n = 127) having no major protocol deviations. Baseline characteristics are shown in Table 1. In all, 337 participants (EE/GSD, n = 167; EE/NGMN, n = 170) completed the study. Mean (± SD) compliance was 99.2% (± 3.7%) with the EE/GSD patch and 99.4 (± 3.5%) with the EE/NGMN patch. 

### Pharmacokinetic analysis (Study 1 only) 

Over the treatment period, mean EE concentrations reached steady state at day 7 during all three treatments. As expected, peak serum EE concentrations were identical for treatments P and E. Maximum EE concentration was higher for treatment M (COC) compared with treatment P (EE/GSD patch) and treatment E (EE/GSD patch with extended application phase). Average serum EE concentrations were similar during each treatment, with a trend towards slightly lower average concentrations for treatment E. Unbound GSD trough concentrations also reached steady state at day 7 during all three treatments. Peak serum concentrations of unbound GSD were again identical for treatment P and treatment E, and ~ 1.7-fold higher for treatment M. Average serum concentrations were slightly lower for treatment E compared with treatment P. Average concentrations after treatment M (COC) were slightly higher compared with treatment P (Table 2). 

Mean total GSD trough concentrations during treatment P reached steady state after 2 – 3 weeks. During treatment M and treatment E, total GSD trough concentrations reached a plateau at day 14. Maximal mean serum concentrations of total GSD were similar during treatment P (6.82 µg/L, CV 49.4%) and treatment E (6.49 µg/L, CV 42.4%), and were lower than during treatment M (10.3 µg/L, CV 29.3%). Average serum total GSD concentrations were relatively similar across the treatments (5.47 µg/L, CV 48.0%; 5.95 µg/L, CV 37.5%; and 4.86 µg/L, CV 33.9%; treatments P, M, and E, respectively). 

With treatment P, mean serum SHBG concentrations increased from pre-dose to day 14, increasing only slightly thereafter to reach steady state on day 28. Following pill intake (treatment M), SHBG concentrations reached a steady state after 2 weeks of intake. The increase in mean SHBG serum concentrations during treatment E showed a similar time course to treatment P, reaching a plateau between days 14 and 21. 

Systemic exposure of EE after application of the patch (AUC_0–168_/7) was in the same range as that after administration of a COC containing 0.02 mg EE (AUC_0–24_), resulting in an AUC ratio for treatment P vs. treatment M of 1.06 (CI, 0.964 – 1.16%), which clearly fulfills bioequivalence criteria (Table 3). 

Due to potentially different induction of the GSD-binding protein SHBG, the concentration of unbound GSD, rather than total GSD, was used to assess the relative bioavailability of GSD. Daily systemic exposure to unbound GSD with treatment P was slightly lower than with treatment M. The mean systemic exposure of GSD, based on the concentration of unbound GSD, was lower for treatment P (82%) than for treatment M. 

Based on these data, the mean daily dose of EE and GSD delivered from the EE/GSD patch was calculated to result in the same systemic exposure as the daily oral administration of 0.0212 mg EE (90% CI: 0.0193 – 0.0233 mg) and 0.0615 mg GSD (unbound; 90% CI: 0.057 – 0.0664 mg). 

### Population pharmacokinetic analysis (study 2 only) 

In the population PK models EE and EE/SHBG/GSD, EE serum concentrations were described with a three-compartment model where the volume of the second peripheral compartment was set to that of the central compartment. A two-compartment model was considered not to be sufficient for a good description of the data, but the data did not allow the estimation of the volume of the second peripheral compartment. A time-dependent release rate from the patch compartment to the skin compartment and a first-order absorption rate from the skin compartment to the central compartment were used to describe EE release and absorption. Inter-individual variability (IIV) was identified for the clearance of EE. 

GSD serum concentrations were described with a two-compartment model with binding to albumin (assumed to be constant) and SHBG in the central compartment, from which only unbound GSD could be eliminated or transported to the peripheral compartment. For EE, only total concentrations were considered since total EE concentrations are not dependent on SHBG concentrations (EE only binds to albumin), and the fraction unbound does not change in relation to the total EE concentration. Additionally, elimination of GSD from the central compartment was described by non-linear Michaelis-Menten elimination. Release and absorption of transdermally administered GSD was described as for EE. IIV was identified for a parameter in the non-linear Michaelis-Menten elimination. A correlation was identified between this parameter and the clearance of EE. 

Serum concentrations of endogenous SHBG were described using a one-compartment model. SHBG concentrations were indirectly up-regulated by EE and down-regulated by GSD. The effect of EE and GSD on SHBG concentrations was delayed using a single-delay compartment. IIV was identified for the SHBG baseline parameter. In all cases, a log-normal distribution for the IIV and a proportional residual error model were assumed. 

**Figure 1:**
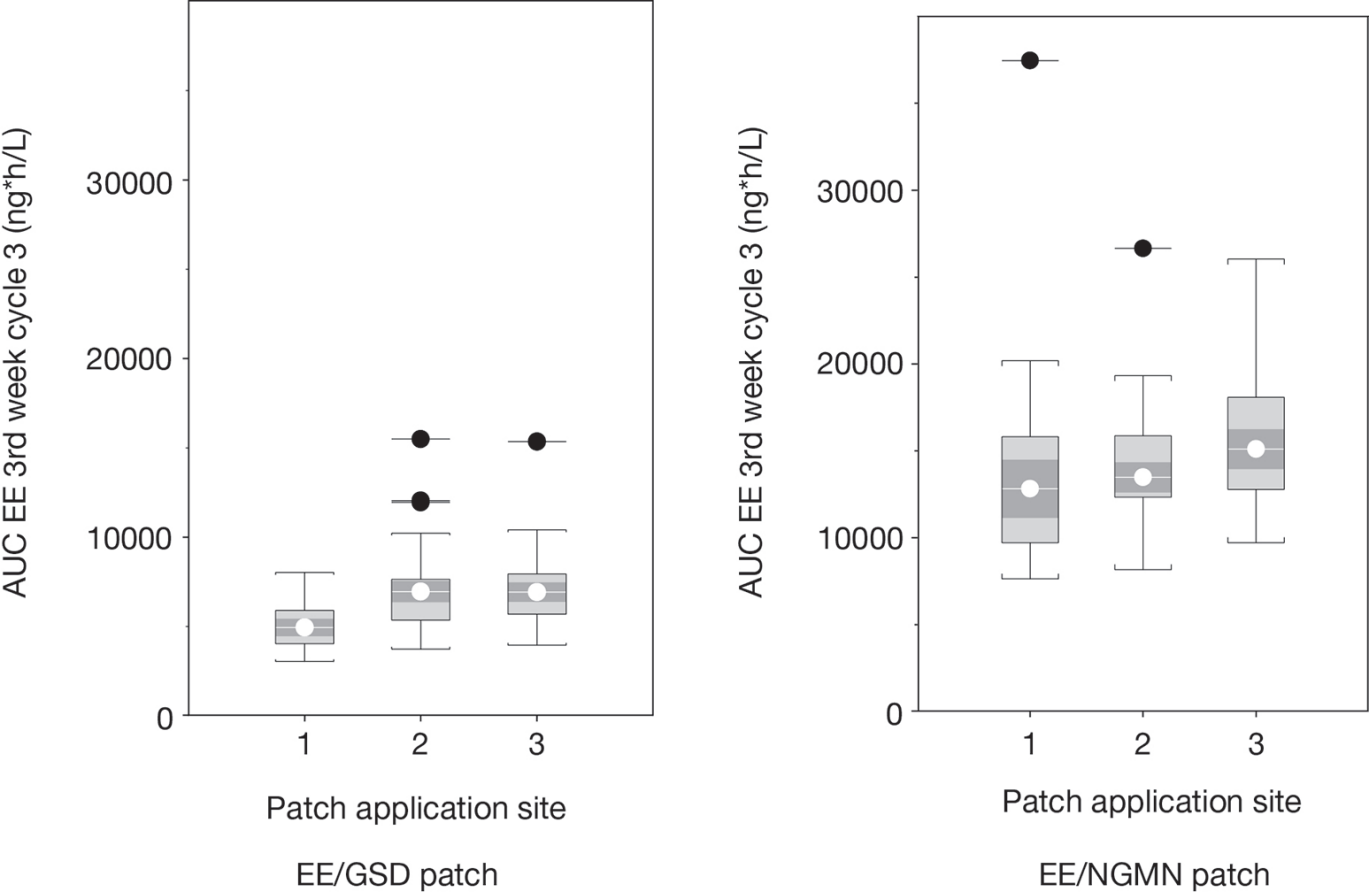
Box plots of AUC_0–168,ss_ for EE in treatment cycle 3 of study 2, shown per treatment and patch application site (1: abdomen, 2: arm, 3: buttocks), analyzed using the “population PK model EE”. AUC_0–168,ss_ = area under the concentration-time curve at steady state; EE = ethinyl estradiol; PK = pharmacokinetic.

In the population PK model EE, an application-site effect was found for the EE/GSD patch: a lower release parameter was estimated for abdomen compared with arm or buttocks. For the EE/NGMN patch, only one release parameter for all application sites was kept since a strong overlap in CIs of the release parameter estimates per application site could be observed, even though comparison of the geometric means of AUC_0–168,ss_, C_av,ss_, and C_max,ss_ suggested a similar application-site effect for the EE/NGMN patch. This is probably due to the high variability of EE after EE/NGMN patch application compared with that for the EE/GSD patch (Figure 1). In the “population PK model EE/SHBG/GSD”, no additional application-site effect was found. Moreover, it was found that the effect of body weight on EE clearance was of borderline statistical significance (0.01 < p < 0.001). Clearance values showed a log-linear increase with body weight (range: 44 – 86 kg) and, for the 5^th^ (49 kg) and 95^th^ (79.9 kg) percentiles of the body weight distribution, EE clearance values were 91% and 113% of the typical clearance (at median weight of 60 kg), respectively. 

**Figure 2:**
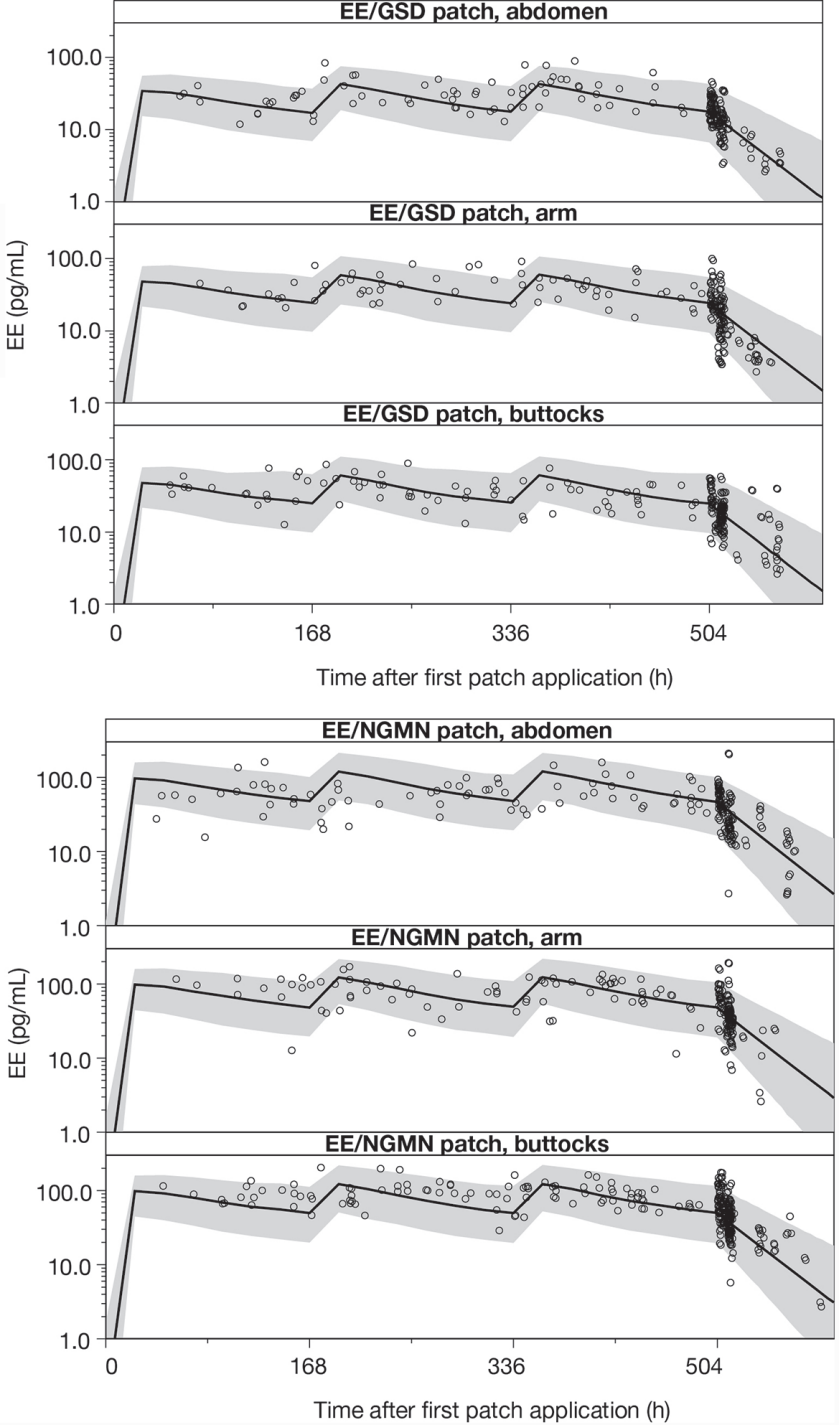
Observed and model-based simulated serum EE concentrations over time, as measured in study 2 and following application to each of the three possible sites. Circles: observed serum concentrations from treatment cycle 3 onwards; black solid line: median of simulations (treatment cycle 3 and 7 combined) based on the “population PK model EE”; gray area: 90% prediction interval based on the “population PK model EE”. EE = ethinyl estradiol; PK = pharmacokinetic.

**Figure 3:**
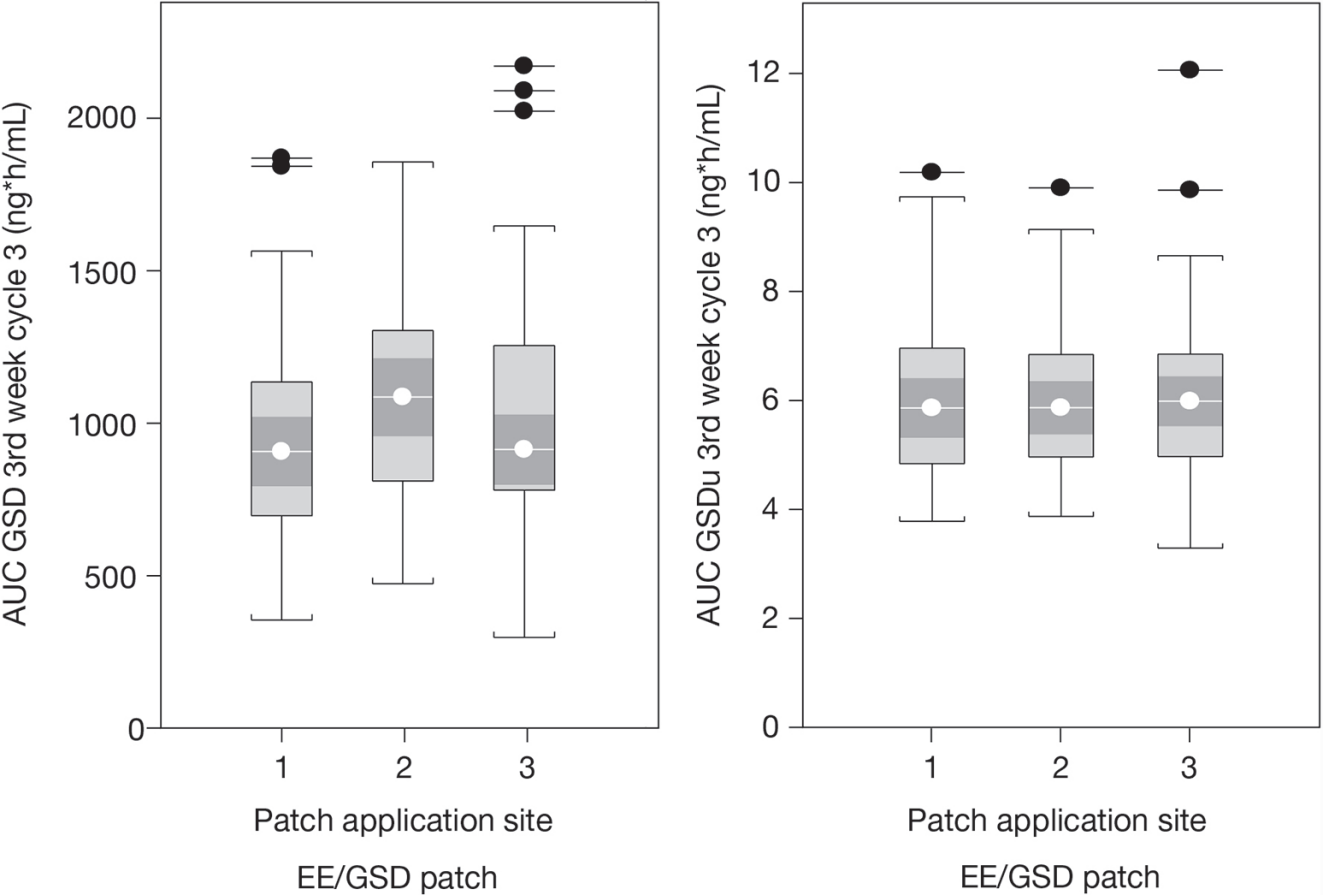
Box plots of steady-state AUC_0–168,ss_ of total GSD and unbound GSD in treatment cycle 3 of study 2, shown per patch application site (1: abdomen, 2: arm, 3: buttocks), analyzed using the “population PK model EE/SHBG/GSD” and actual application sites (determined as used at ≥ 50% of the respective treatment cycle). AUC_0–168,ss_ = area under the concentration-time curve at steady state; EE = ethinyl estradiol; GSD = gestodene; GSDu = unbound gestodene; PK = pharmacokinetic; SHBG = sex hormone-binding globulin.

The “population PK model EE” was used to estimate individual steady-state PK parameters for the 3^rd^ week of treatment cycles 1, 3, and 7, and showed that AUC_0–168,ss_, C_av,ss_, and C_max,ss_ values for EE were greater with the EE/NGMN patch than with the EE/GSD patch for all three application sites (Table 4). These data are also representative of treatment cycles 1 and 7, as steady state was effectively achieved by week 3 in all treatment cycles (data not shown). Observed and simulated serum EE concentrations over time for the three application sites are shown in Figure 2. The population PK model for EE, GSD, and SHBG showed that steady state was achieved for all PK parameters by week 3 in all treatment cycles. For EE, AUC_0–168,ss_, C_av,ss_ and C_max,ss_ values for EE tended to be greater for patches applied to the arm and buttock compared with those applied to the abdomen. There were no marked differences between application sites for total and unbound GSD or SHBG (Table 5) (Figure 3). 

### Safety results 

In study 1, 23 out of 25 women experienced at least one treatment-emergent AE (TEAE); in 21 women these were drug related. Three women prematurely discontinued study participation due to a TEAE (mild psycho-vegetative syndrome, moderate gastroenteritis, and moderate allergic contact eczema during treatment E; the case of eczema was assessed as related to study drug). The most frequent TEAEs were headache, application-site reaction, and nasopharyngitis. All AEs were mild to moderate in severity, and all were resolved. There were no serious AEs or deaths. 

In study 2, 95 women (47.5%) using the EE/GSD patch and 79 (39.9%) using the EE/NGMN patch reported at least one TEAE during the study. Drug-related AEs were reported by 24.0% of women using the EE/GSD patch and 20.7% using the EE/NGMN patch. With the EE/GSD patch, the most common treatment-related AEs were application-site reaction (7.5%), application-site pruritus (5.5%), cervical dysplasia (5.5%), and metrorrhagia (4.0%). In the EE/NGMN group, the most common events were headache (4.0%), application-site reaction (4.0%), metrorrhagia (4.0%), and breast pain (4.0%). Serious TEAEs were reported by 6 women; however, none was considered related to study medication. No deaths were reported. 

## Discussion 

The pharmacokinetic parameters and statistical evaluation of study 1 show that, during steady state, the average daily AUC for EE after oral intake of a COC containing 0.02 mg EE and 0.075 mg GSD (treatment M) is the same as the average daily AUC during the 7-day dermal application of EE/GSD patch (treatment P). The mean AUC ratio of treatment P vs. treatment M for EE after patch application (Table 3) was clearly in the range of bioequivalence acceptance limits. The calculated EE dose of 0.02 mg does, however, not reflect the real amount released from the patch because EE is not completely bioavailable after oral administration. Based on data from several clinical studies, the mean (range) oral bioavailability of EE is ~ 45 (38 – 48)% [15]. The real amount of EE released from the patch is therefore around half the amount determined in the present study because no pre-systemic metabolism in the skin is expected after transdermal administration. 

Based on the average daily AUC of unbound GSD, the amount of GSD released by the EE/GSD patch during the 7-day wearing period resulted in a systemic exposure similar to that after daily oral administration of a GSD dose of ~ 0.06 mg (mean AUC ratio patch vs. 0.075 mg GSD COC was 82%). As GSD is almost completely bioavailable after oral administration [16, 17], the GSD dose determined in the present study reflects the real amount released after dermal application of the patch. 

Although the EE/GSD patch and the EE/NGMN patch assessed in study 2 contain similar amounts of EE (0.55 mg and 0.6 mg, respectively), the findings of this study show that systemic exposure to EE with the EE/GSD patch is approximately half (or less) than that seen with the EE/NGMN patch. This applies to all three application sites. Other studies with the EE/NGMN patch reported lower EE exposure than reported in the current study for the direct comparison between the EE/GSD patch and the EE/NGMN patch [18, 19]. These concentrations have been determined after single patch application over 7 days and not during true steady state as in the current study, where concentrations of EE are generally higher than after single application. The systemic exposure to EE with the EE/GSD patch in this comparative study was, however, still clearly lower than that previously measured for the EE/NGMN patch. 

Exposure of EE with the EE/GSD patch when applied for 7 days was also lower than that reported in a recent study describing the PK parameters of a patch containing EE and levonorgestrel [20]. This comparatively low exposure to EE may be important, since EE exposure appears to be positively related to the risk of developing venous thromboembolism [21]. Furthermore, the lower EE exposure with the EE/GSD patch may also have a positive impact on other AEs, such as EE-related breast pain, which was experienced more often by participants wearing the EE/NGMN patch compared with those wearing the EE/GSD patch. In addition, in the current study, cycle control was not affected by lower EE exposure for the EE/GSD patch compared with the contraceptive patch containing a higher dose of EE (Gruber et al., manuscript in preparation). 

Our results also show that EE exposure with the EE/GSD patch was bioequivalent to that which was seen with a currently available reference COC containing a combination of 0.02 mg EE and 0.075 mg GSD, while unbound GSD exposure was slightly lower. In addition, there was negligible influence from demographic covariates, and although body weight had a small but borderline significant effect on EE clearance, this was considered unlikely to be clinically meaningful. 

## Conclusion 

The EE/GSD patch resulted in a reduced exposure to EE compared with the EE/NGMN transdermal contraceptive patch, and in the same systemic exposure that would be achieved by the oral administration of a COC containing 0.02 mg EE and 0.06 mg GSD. Prolonged wearing of the patch for up to 10 days did not result in a distinct decline in EE or GSD serum concentrations. In addition, the EE/GSD patch also had a good safety profile. These findings, set alongside those of previous studies, provide further support for the EE/GSD patch as an effective and well-tolerated alternative to both currently available transdermal contraceptives and COCs containing EE and GSD. 

## Acknowledgments 

The population PK analysis was performed under supervision of Bayer Pharma AG (Berlin, Germany) by Maurice Ahsman and Jean Smeets, Leiden Experts on Advanced Pharmacokinetics and Pharmacodynamics, Leiden, The Netherlands. Statistical analyses were performed by Matthias Ludwig, Bayer Pharma AG. Editorial assistance was provided by Ogilvy 4D and was funded by Bayer Pharma AG. 

## Conflict of interest 

All authors are employees of Bayer Pharma AG. 

**Table 1:** Baseline demographics of women randomized in studies 1 and 2, shown as mean ± SD (range).

Study 1	Sequence P–M–E* n = 13	Sequence M–P–E^† ^ n = 12		Total n = 25
Age, years	27.2 ± 6.1 (19 – 37)	29.2 ± 7.0 (19 – 39)		28.1 ± 6.5 (19 – 39)
Weight, kg	68.3 ± 10.4 (57.4 – 88.2)	64.7 ± 9.3 (49.5 – 81.4)		66.6 ± 9.8 (49.5 – 88.2)
Height, cm	167.6 ± 5.7 (155 – 177)	164.8 ± 7.1 (148 – 175)		166.2 ± 6.5 (148 – 177)
BMI, kg/m^2^	24.3 ± 3.1 (19.9 – 29.8)	23.8 ± 2.8 (19.6 – 30.3)		24.1 ± 2.9 (19.6 – 30.3)

Study 2	EE/GSD patch^† ^ n = 200		EE/NGMN patch^†† ^ n = 198	
Age, years	24.9 ± 4.3 (18 – 35)		24.5 ± 4.6 (18 – 35)	
Weight, kg	61.2 ± 9.2 (45 – 89)		63.3 ± 9.0 (42 – 89)	
Height, cm	167.3 ± 5.9 (152 – 185)		167.9 ± 5.9 (150 – 184)	
BMI, kg/m^2^	21.8 ± 2.8 (17 – 30)		22.4 ± 2.8 (17 – 30)	

*Sequence P–M–E = treatment P: 0.55 mg EE + 2.1 mg GSD per patch (4 × 7-day wearing period); treatment M: 0.02 mg EE + 0.075 mg GSD per pill (21-day, oral, once daily); treatment E: 0.55 mg EE + 2.1 mg GSD per patch (2 × 7-day, followed by 1 × 10-day, wearing period); ^†^Sequence M–P–E = treatment M: 0.02 mg EE + 0.075 mg GSD per pill (21-day, oral, once daily); treatment P: 0.55 mg EE + 2.1 mg GSD per patch (4 × 7-day wearing period); treatment E: 0.55 mg EE + 2.1 mg GSD per patch (2 × 7-day, followed by 1 × 10-day, wearing period); ^†^0.55 mg EE/2.1 mg GSD; ^††^0.6 mg EE/6 mg NGMN; the only commercially available patch in study countries. BMI = body mass index; EE = ethinyl estradiol; GSD = gestodene; SD = standard deviation.

**Table 2:** Mean PK parameters for EE (upper table) and unbound GSD (lower table) in study 1.

		Treatment P* (n = 22)	Treatment M^† ^ (n = 22)	Treatment E^‡§ ^ (n = 20)
PK parameter	Unit	Geometric mean (CV)		
C_max_	ng/L	37.5 (24.4%)	59.6 (34.7%)	37.8 (24.6%)
t_max (min, max)_	h	35.0 (12.0, 72.2)	1.5 (0.5, 3.0)	24.8 (11.9, 48.0)
AUC_0–168_/7	ng×h/L	648 (22.4%)	–	–
AUC_0–24_	ng×h/L	–	611 (30.6%)	–
t_1/2_	h	22.9 (20.1%)^¶^	17.6 (23.0%)^#^	22.7 (20.3%)**
C_min_	ng/L	17.9 (27.8%)	12.9 (41.3%)	15.6 (26.0%)^††^
C_av_	ng/L	27.0 (22.4%)	25.5 (30.6%)	23.9 (23.5%)^††^
AUC_0–168_	ng×h/L	4,533 (22.4%)	–	–

		Treatment P* (n = 22)	Treatment M^† ^ (n = 22)	Treatment E^‡§ ^ (n = 20)
PK parameter	Unit	Geometric mean (CV)		
C_max_	µg/L	0.0485 (34.6%)	0.0844 (19.8%)	0.0496 (35.9%)
t_max (min, max)_	h	36.1 (24.9, 71.8)	1 (0.5, 1.5)	35.7 (23.5, 95.6)
AUC_0–168_/7	µg×h/L	0.910 (32.7%)	–	–
AUC_0–24_	µg×h/L	–	1.11 (22.4%)	–
t_1/2_	h	27.1 (22.9%)	25.2 (18.8%)^††^	27.3 (23.7%)
C_min_	µg/L	0.0270 (32.9%)	0.0317 (28.0%)	0.0217 (32.5%)
C_av_	µg/L	0.0379 (32.7%)	0.0462 (22.4%)	0.0339 (30.6%)
AUC_0–168_	µg×h/L	6.37 (32.7%)	–	–

*Treatment P: 0.55 mg EE + 2.1 mg GSD per patch (4 × 7-day wearing period); ^†^treatment M: 0.02 mg EE + 0.075 mg GSD per pill (21-day, oral, once daily); ^‡^treatment E: 0.55 mg EE + 2.1 mg GSD per patch (2 × 7-day, followed by 1 × 10-day, wearing period); ^§^Two women dropped out in period 3; ^¶^Data from 6 participants; ^#^Data from 18 participants; **Data from 5 participants; ^††^Data from 20 participants. AUC_0–168_ = area under the concentration-time curve from time point of 4^th^ (last) patch application until its removal 1 week later (treatment P); AUC_0–168_/7 = area under the concentration-time curve from time point of 4^th^ (last) patch application until its removal 1 week later (treatment P) divided by 7; AUC_0–24_ = area under the concentration-time curve from time point of 21^st^ (last) pill administration until 24 hours thereafter; C_av_ = average concentration obtained during last application interval; C_max_ = maximum (peak) serum concentration obtained during last application interval; C_min_ = minimum serum concentration during last application interval; CV = coefficient of variation; EE = ethinyl estradiol; GSD = gestodene; PK = pharmacokinetic; t_1/2_ = half-life associated with terminal slope; t_max_ = time to reach maximum (peak) concentration in serum obtained during last application interval.

**Table 3:** Mean daily area under the concentration-time curve (AUC) ratio and estimated daily dose delivery for EE and GSD after EE/GSD patch application (treatment P) compared with those for oral administration of a pill containing EE and GSD (treatment M).

PK parameter	Analyte	Treatments	Geometric mean AUC ratio of treatment P* vs. treatment M^†^	Lower 90% confidence limit	Upper 90% confidence limit	Geometric mean (range) estimated daily dose delivery µg
AUC^‡^	GSD unbound	P vs. M	0.820	0.760	0.885	61.5 (57.0 – 66.4)
AUC^‡^	EE	P vs. M	1.06	0.964	1.16	21.2 (19.3 – 23.3)

*Treatment P: 0.55 mg EE + 2.1 mg GSD per patch (4 × 7-day wearing period); ^†^treatment M: 0.02 mg EE + 0.075 mg GSD per pill (21-day, oral, once daily); ^‡^AUC_0–168_/7 for treatment P and AUC_0–24_ for treatment M. AUC_0–168_ = area under the concentration-time curve from time point of 4^th^ (last) patch application until its removal 1 week later (treatment P); AUC_0–24_ = area under the concentration-time curve from time point of 21^st^ (last) pill administration until 24 hours thereafter; EE = ethinyl estradiol; GSD = gestodene; PK = pharmacokinetic.

**Table 4:** Geometric mean (CV %) PK parameters for EE after application of the EE/GSD patch and the EE/NGMN patch, by application site in study 2. Parameters were estimated using the population PK model EE for the third week of treatment cycle 3 (also representative of treatment cycles 1 and 7 because steady state was effectively achieved by week 3 in all treatment cycles).

Application site	Parameter	Unit	EE/GSD patch*	0.6 mg EE/6 mg NGMN patch	0.6 mg EE/6 mg NGMN patch: EE/GSD patch
Abdomen			n = 37	n = 33	
	AUC_0–168,ss_	ng×h/L	4,971 (26.1%)	12,739 (33.4%)	2.6
	C_av,ss_	ng/L	29.6 (26.1%)	75.8 (33.4%)	2.6
	C_max,ss_	ng/L	43.7 (18.2%)	117 (23.0%)	2.7
Arm			n = 37	n = 41	
	AUC_0–168,ss_	ng×h/L	6,780 (30.8%)	13,839 (24.4%)	2.0
	C_av,ss_	ng/L	40.4 (30.8%)	82.4 (24.4%)	2.0
	C_max,ss_	ng/L	60.4 (21.5%)	123 (16.9%)	2.0
Buttocks			n = 42	n = 53	
	AUC_0–168,ss_	ng×h/L	6,845 (28.2%)	15,335 (24.3%)	2.2
	C_av,ss_	ng/L	40.7 (28.2%)	91.3 (24.3%)	2.2
	C_max,ss_	ng/L	60.7 (19.6%)	132 (17.3%)	2.2

*EE/GSD patch: 0.55 mg EE/2.1 mg GSD. AUC_0–168,ss_ = area under the concentration–time curve at steady state; C_av,ss_ = average serum concentration at steady state; C_max,ss_ = maximum serum concentration at steady state; CV = coefficient of variation; EE = ethinyl estradiol; GSD = gestodene; NGMN = norelgestromin; PK = pharmacokinetic.

**Table 5:** Geometric mean (CV %) PK parameters for EE, SHBG, and total and unbound GSD for the EE/GSD patch (0.55 mg EE/2.1 mg GSD) per application site in study 2, estimated using the population PK model EE/SHBG/GSD for the third week of treatment cycle 3 (also representative of treatment cycles 1 and 7 because steady state was effectively achieved by week 3 in all treatment cycles).

Analyte	Parameter	Unit	Abdomen (n = 37)	Arm (n = 37)	Buttocks (n = 42)
EE	AUC_0‒168,ss_	(ng×h)/L	4,991 (25.5%)	6,746 (29.8%)	6,818 (28.3%)
	C_av,ss_	ng/L	29.7 (25.5%)	40.2 (29.8%)	40.6 (28.3%)
	C_max,ss_	ng/L	43.6 (18.0%)	59.8 (21.0%)	60.2 (19.8%)
GSD total	AUC_0‒168,ss_	(µg×h)/L	897 (38.3%)	1005 (35.6%)	947 (45.9%)
	C_av,ss_	µg/L	5.34 (38.3%)	5.98 (35.6%)	5.64 (45.9%)
	C_max,ss_	µg/L	6.54 (32.8%)	7.19 (30.7%)	6.83 (38.8%)
GSD unbound	AUC_0‒168,ss_	(µg×h)/L	5.83 (23.9%)	5.92 (25.1%)	5.88 (27.6%)
	C_av,ss_	µg/L	0.0347 (23.9%)	0.0353 (25.1%)	0.0350 (27.6%)
	C_max,ss_	µg/L	0.0434 (18.3%)	0.0434 (20.2%)	0.0435 (20.6%)
SHBG	C_av,ss_	nmol/L	237 (35.6%)	268 (38.1%)	251 (38.3%)

AUC_0–168,ss_ = area under the concentration-time curve at steady state; C_av,ss_ = average serum concentration at steady state; C_max,ss_ = maximum serum concentration at steady state; EE = ethinyl estradiol; GSD = gestodene; PK = pharmacokinetic; SHBG = sex hormone-binding globulin.
